# Silica Modified with Polyaniline as a Potential Sorbent for Matrix Solid Phase Dispersion (MSPD) and Dispersive Solid Phase Extraction (d-SPE) of Plant Samples

**DOI:** 10.3390/ma11040467

**Published:** 2018-03-22

**Authors:** Ireneusz Sowa, Magdalena Wójciak-Kosior, Maciej Strzemski, Jan Sawicki, Michał Staniak, Sławomir Dresler, Wojciech Szwerc, Jarosław Mołdoch, Michał Latalski

**Affiliations:** 1 Department of Analytical Chemistry, Medical University of Lublin, Chodźki 4a, 20-093 Lublin, Poland; maciej.strzemski@poczta.onet.pl (M.S.); 91chem91@gmail.com (J.S.); michal_staniak@wp.pl (M.S.); wojciech.szwerc@onet.eu (W.S.); jmoldoch@iung.pulawy.pl (J.M.); 2 Department of Plant Physiology, Institute of Biology and Biochemistry, Maria Curie-Skłodowska University, Akademicka 19, 20-033 Lublin, Poland; dresler.slawomir@gmail.com; 3 Department of Biochemistry and Crop Quality, Institute of Soil Science and Plant Cultivation, State Research Institute, ul. Czartoryskich 8, 24-100 Puławy, Poland; 4 Children’s Orthopaedics Department, Medical University of Lublin, Gębali 6, 20-093 Lublin, Poland; michall1@o2.pl

**Keywords:** polyaniline, Si-PANI, d-SPE, MSPD, triterpenes, sample pretreatment

## Abstract

Polyaniline (PANI) is one of the best known conductive polymers with multiple applications. Recently, it was also used in separation techniques, mostly as a component of composites for solid-phase microextraction (SPME). In the present paper, sorbent obtained by in situ polymerization of aniline directly on silica gel particles (Si-PANI) was used for dispersive solid phase extraction (d-SPE) and matrix solid–phase extraction (MSPD). The efficiency of both techniques was evaluated with the use of high performance liquid chromatography with diode array detection (HPLC-DAD) quantitative analysis. The quality of the sorbent was verified by Raman spectroscopy and microscopy combined with automated procedure using computer image analysis. For extraction experiments, triterpenes were chosen as model compounds. The optimal conditions were as follows: protonated Si-PANI impregnated with water, 160/1 sorbent/analyte ratio, 3 min of extraction time, 4 min of desorption time and methanolic solution of ammonia for elution of analytes. The proposed procedure was successfully used for pretreatment of plant samples.

## 1. Introduction

Polyaniline (PANI) is one of the best known conductive polymers with broad application in many fields such as chemistry, physics, optics, materials and biomedical science. It was applied, e.g., as a component of sensors, diodes, solar batteries, electromagnetic shields, and materials for protection against corrosion [1,2,3,4,5]. Due to the unique properties of PANI such as simplicity of synthesis, mechanical and chemical flexibility, resistance on pH and temperature, hydrophobicity, π-conjugated structure, polar groups, and ion exchange ability [1,2,6,7], it also proved to be useful in extraction techniques, mainly as a component of various composites, e.g., with graphene [8], montmorillonite [9], cyclodextrin [10], polyester [11], etc. Metal fibers covered with these types of materials were used for solid-phase microextraction (SPME), magnetic solid phase extraction (MSPE), headspace solid phase microextraction (HS-SPME) and magnetic dispersive solid phase extraction (MDSPE) of different target compounds from the matrix. Polyaniline and sorbents modified with PANI were also successfully applied in solid phase extraction (SPE) [12,13,14,15,16,17,18]. However, reports on the applications of polyaniline based materials for plant samples are scarce. For instance, Arnnok et al. [19] used polyaniline-modified zeolite in DSPE of fruit and vegetables to isolate carbamate, organophosphate, sulfonylurea, pyrethroid and neonicotinoid; Alizadeh et al. [11] adapted polyester-polyaniline fiber for SPME of volatile organic compounds (VOCs) from lemon juice, and silica modified with polyaniline (Si-PANI) was applied as an SPE adsorbent for sample clean-up before HPLC analysis of triterpenes in plant extracts [18]. SPE is one of the most common techniques for pretreatment of biological materials; however, it has some drawbacks, e.g., the relatively time-consuming procedure, the high consumption of solvent, and the risk of losses of volatile analytes [20,21,22]. Therefore, recently, the other techniques have gained attention of researchers dispersive solid phase extraction (d-SPE) and matrix solid–phase extraction (MSPD). Both techniques are useful for pretreatment of samples with complex matrix, due to high efficiency combined with low cost, simplicity and high speed of process [23,24,25,26,27].

In the present study, Si-PANI was tested as a sorbent for d-SPE and MSPD of oleanolic, ursolic and betulinic acid from *Viscum album* L. and *Ocimum basilicum* L. Polyaniline was chosen for covering of silica because the mix mode retention mechanism allows to retain various group of analytes, both charged and uncharged, cationic and anionic forms. The extraction conditions such as: form of PANI, washing solution and eluent were optimized experimentally. The physico-chemical features of the sorbent such as polyaniline form and quality the deposition of PANI film on silica were verified by Raman spectroscopy and microscopy combined with automated procedure using computer image analysis.

## 2. Methods and Materials

### 2.1. Materials and Reagents

Silica gel Lichrospher 60 Si, aniline (for analysis EMSURE), ammonium peroxydisulphate (extra pure) used for synthesis of the adsorbent, solvents and reagents: ammonia solution, hydrochloric acid, ortho-phosphoric acid, ammonium acetate, methanol, acetonitrile (gradient grade for liquid chromatography) were from Merck (Darmstadt, Germany). Water was deionized using ULTRAPURE Milipore Direct-Q^®^ 3UV–R (Merck). Standards of betulinic (BA) (≥98%), oleanolic (OA) (≥97%), and ursolic (UA) (≥98.5%) acid were purchased from Sigma-Aldrich (St. Louis, MO, USA).

Extracts from *Viscum album* L. and *Ocimum basilicum* L were prepared by extraction of pulverized plant material (1.00 g) with methanol (2 × 20 mL) in ultrasonic bath (2 × 15 min). The obtained extracts were concentrated to 10 mL and filtered through Millex Samplicity Filters 0.20 μm (Merck).

### 2.2. Methodology

#### 2.2.1. Synthesis and Characteristic of Si-PANI Sorbent

In situ polymerization of aniline was conducted directly on silica particles with the use of ammonium peroxydisulphate as an oxidation agent at a temperature of 0–2 °C. The procedure was described in detail in our previous publications [7,28]. The deposition of polyaniline on silica particles was verified by Raman analysis using a Thermo Scientific DXR confocal Raman microscope with the Omnic 8 software (Thermo Fisher Scientific Madison, Madison, WI, USA). The parameters for analysis of PANI distribution were as follows: excitation laser wavelength 780 nm, filters 780 nm, registered wavenumber range from 200 to 2000 cm^−1^, laser power 10 mW and exposure time to 5 s per point.

Video images of silica and Si-PANI obtained with the use of confocal Raman microscope (50× magnification) were binarized and segmented using Fiji image processing and ImageJ software analysis [29,30]. The dimensions of analyzed beads were calculated on the basis of Feret diameter [31] using GrapPad Prism 5.0 (GrapPad Software, San Diego, CA, USA).

Si-PANI was deprotonated or protonated using 0.1 M methanolic solution of ammonia and HCl, respectively and the obtained sorbents were pre-washed with methanol (5 mL) and water (5 mL) to neutral pH of leakage.

#### 2.2.2. Extraction Experiments

Optimisation of extraction procedure was conducted using a methanolic standard solution of oleanolic (OA), betulinic (BA) and ursolic acid (UA) at concentration of 0.1 mg/mL. All the experiments were performed in triplicate at ambient temperature.

##### Dispersive Solid Phase Extraction (d-SPE)

OA, UA and BA solution (1 mL) was mixed with various amounts of sorbent (100, 150, 200 and 250 mg), degassed, and dynamically shaken using vortex for 1–10 min. The suspension was subsequently centrifuged at 9000 rpm for 5 min, supernatant was removed and analyzed with the use of HPLC. The amount of retained compounds was calculated as a difference between applied amount and amount found in supernatant.

The analytes were eluted by shaking with 3 mL portions of various solvents, supernatants were filtered, analyzed with the use of HPLC and % of recovery was calculated.

Finally, the optimized d-SPE conditions were applied for sample pretreatment of plant extracts.

#### 2.2.3. Matrix Solid Phase Dispersion (MSPD)

Pulverized plant material was mixed with Si-PANI, ground to powder (ca. 5 min), packed into a 3-mL polypropylene column, and retained by two polyethylene frits. Eluent was passed through the column using Millipore vacuum pump system (Merck) at the flow rate of 1 mL/min and the eluates were analyzed by HPLC.

#### 2.2.4. HPLC Analysis

The HPLC analysis was conducted using a VWR Hitachi Chromaster 600 chromatograph with a spectrophotometric detector (DAD) and EZChrom Elite software (Merck) on a Discovery C18 reversed-phase column (25 cm × 4.0 mm i.d., 5 μm particle size) (Supelco, Sigma-Aldrich, St. Louis, MO, USA). Mobile phase consisted of acetonitrile-water—1% phosphoric acid (90:10:0.5 *v*/*v*/*v*). Flow rate of eluent was 1 mL/min and column temperature was 10 °C [18]. Chromatograms were recorded from 200 to 400 nm. The triterpenic acids were quantified at 205 nm.

## 3. Results and Discussion

### 3.1. Characteristics of Si-PANI

**Figure 1:**
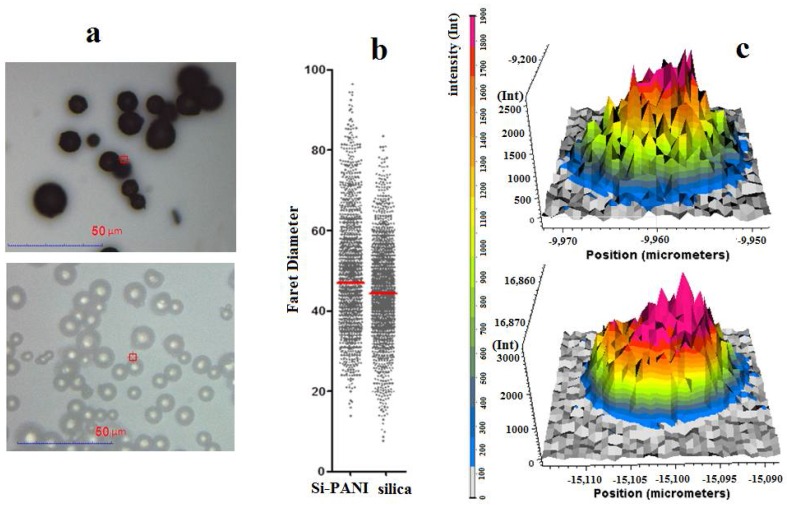
The morphology of Si-PANI particles: (**a**) microscope picture of silica and Si-PANI; (**b**) particle diameter distribution of silica and Si-PANI; (**c**) the examples of spatial distribution of polyaniline on the surface within the particle.

The morphology of Si-PANI particles was assessed using a confocal microscope and automated procedure of computer image analysis (Figure 1). Blue color of Si-PANI particles showed that polyaniline was successfully deposited on silica (Figure 1a). Moreover, no changes of particle shape were observed and this proved that the synthesis conditions did not cause the destruction of silica. The analysis of diameters (Figure 1b) showed the slight increase of Si-PANI particle diameter (average diameter was 10.7 µm) comparing to bare silica (average diameter was 10 µm) as a result of covering the surface with polyaniline film. The intensity of the PANI signal recorded during Raman analysis proved that polyaniline was deposited more intensively inside the adsorbent grain (Figure 1c). This may be explained by the fact that silica has a porous structure and the surface of grain inner pores is larger than the outside pores.

**Figure 2:**
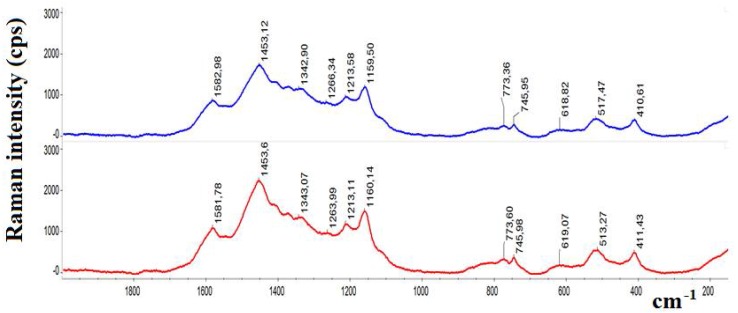
Smoothed Raman spectra of Si PANI sorbent: protonated (red line) and deprotonated (blue line).

Polyaniline may occur in various forms [1]; therefore, in order to establish its form after protonation and deprotonation of Si-PANI bed, the Raman spectra were recorded (Figure 2). Both spectra matched the spectral pattern of the emeraldine [32]. Although minor shifts in some peak positions were observed, the differences between spectra were irrelevant.

### 3.2. Optimization of d-SPE Parameters

In order to establish the optimal conditions for d-SPE of triterpenic acids, the main parameters affecting the extraction efficiency and recovery of analytes were investigated using standard solution of UA, OA and BA.

#### 3.2.1. Form of PANI and Impregnation Solution

**Figure 3:**
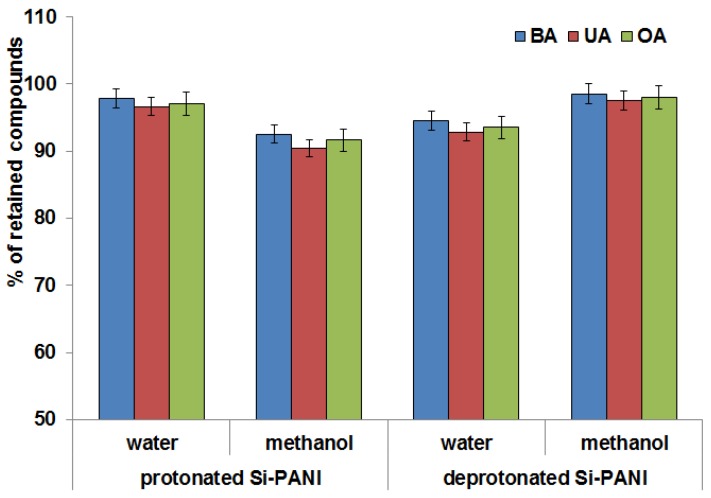
Percentage of retained analytes depending on Si-PANI form and impregnation solution.

The interaction of sorbent with analyte strongly affect the ability of trapping the analyte from solution. Four variants of experiment, using protonated (Si-PANI (+)) and deprotonated (Si-PANI) sorbent impregnated with methanol or water, were conducted to establish the optimal conditions to bond the highest amount of investigated compounds. The results are presented in Figure 3.

As can be seen, the form of polyaniline and impregnation have an impact on efficiency of trapping the triterpenic acids from solution. Surprisingly, a high percentage of extraction efficacy was obtained both for protonated and deprotonated sorbent; however, the different impregnation solutions were required for particular form (water and methanol, respectively).

**Figure 4:**
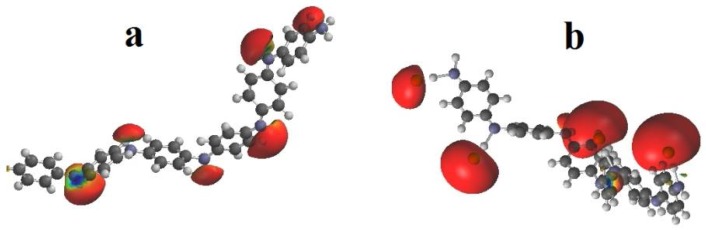
Modeled chain of polyaniline: (**a**) Deprotonated and (**b**) Protonated form.

The structure of Si-PANI and Si-PANI (+) was modeled (Figure 4) to compare the charge density of the surface what could be helpful to explain the observed effect.

Based on modeled structures, significant differences in distribution of charge on polyaniline were noted. As a result, the protonation of PANI with HCl, surface of sorbent was positively charged, the anions (Cl^−^) were accumulate to compensate and the electrical double layer was formed (on Figure 4b the density of charge at Cl^−^ is visible). Presumably, Si-PANI (+) gained the ability to anion exchange. Impregnation with water occurred optimal for Si-PANI (+) because in water the ionization of analytes increased and then, the ability to exchange Cl^-^ on anionic analytes was possible (ion exchange mode of retention).

In turn, on deprotonated Si-PANI, the charges were focused on nitrogen (Figure 4a) and the retention was probably mostly caused by π-π interactions between aromatic rings of analytes and aromatic rings of sorbent which were enhanced by methanol [33].

#### 3.2.2. Time of Extraction

**Figure 5:**
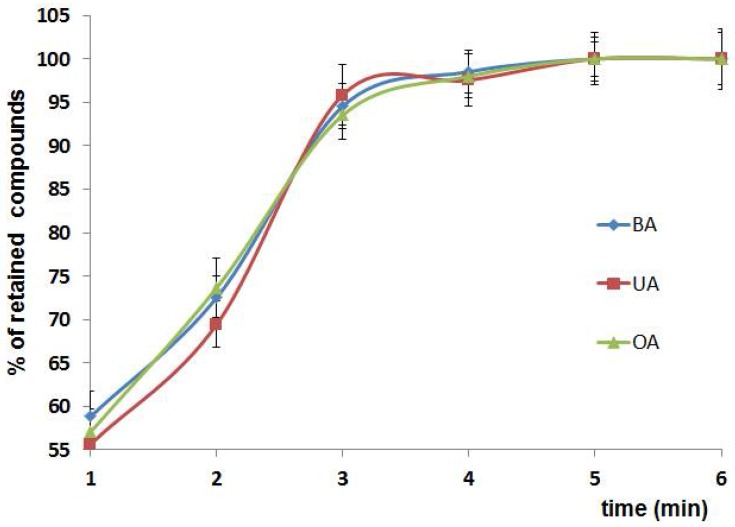
Effect of extraction time on percentage of retained analytes.

The bonding of analyte in d-SPE strongly depends on time. The partition of analyte between solution and sorbent is dynamic process and appropriate time is necessary to obtain equilibrium [26,27]. Our investigation showed that the amount of bonded triterpenic acids increased up to 3 min and then remained constant (the plateau effect was observed) (Figure 5). No statistically significant differences between the investigated compounds or between both forms of sorbent were noted.

#### 3.2.3. Ratio of Sorbent to Analyte

**Figure 6:**
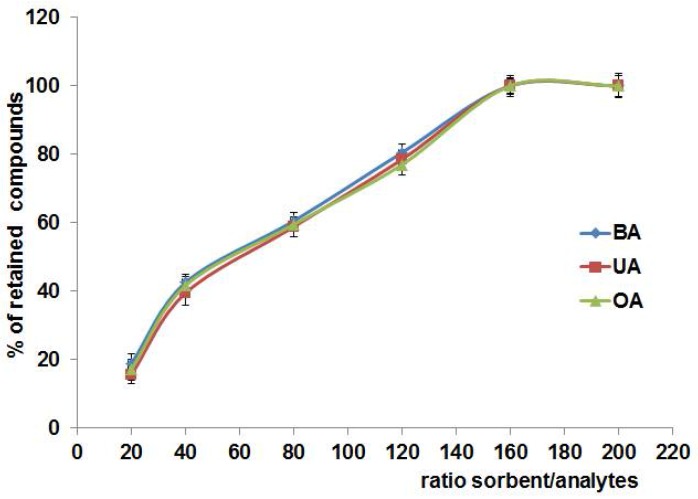
Effect of sorbent/analytes ratio on percentage of retained analytes.

Since the number of active sites on adsorbent surface should be sufficient to trap the total amount of target compound, the sorbent/analyte ratio is a significant factor affecting the extraction efficiency. As can be seen on Figure 6 the ratio 160:1 (mg of sorbent/mg analytes) was found to be optimal for all investigated triterpenic acids. Moreover, we noticed that the curves of relationship between sorbent/analyte ratio and percentage of retained compound were similar for protonated and deprotonated Si-PANI.

#### 3.2.4. Elution Solvent

The high recovery of analytes bonded with sorbent requires the selection of appropriate desorption solvent. Different factors should be taken into consideration, e.g., affinity of solvent to sorbent and analyte, its volatility and solubility of target compound.

**Figure 7:**
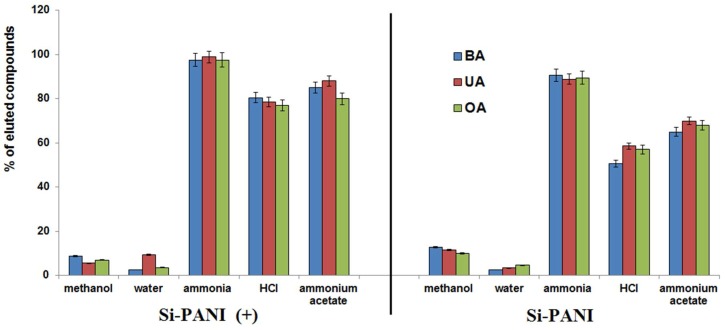
Effect of various solutions on the elution of retained analytes.

Organic solvents with relatively low boiling point such as methanol, acetonitrile, ethanol and acetone are the most commonly used because they easily evaporate and therefore, the concentration of sample solution is possible without the risk of degradation the analyte. Sometimes, acids or buffers are added to change the ionization of investigated components or adsorbent and hereby, to decrease their interaction. As our study showed, the pure solvents had weak elution strength toward triterpenic acids bonded on Si-PANI. However, the additional low amount of ammonia, hydrochloric acid and ammonium acetate significantly increased the ability to elute of investigated triterpenes, probably as a result of ion exchange of bonded analyte on anion from mobile phase. The highest elution strength had 0.1 M methanolic solutions of investigated modifiers (Figure 7). The differences of elution from Si-PANI and Si-PANI (+) could be explain by stronger π-π interaction on deprotonated form. The higher % of recovery was obtained for protonated form of adsorbent (above 97%, 77%, and 80% for ammonia, HCl and ammonium acetate solution, respectively) comparing to deprotonated (ca. 89%, 55%, and 67% for ammonia, HCl and ammonium acetate solution, respectively).

Therefore, Si-PANI (+) was chosen for further experiments.

#### 3.2.5. Desorption Time

**Figure 8:**
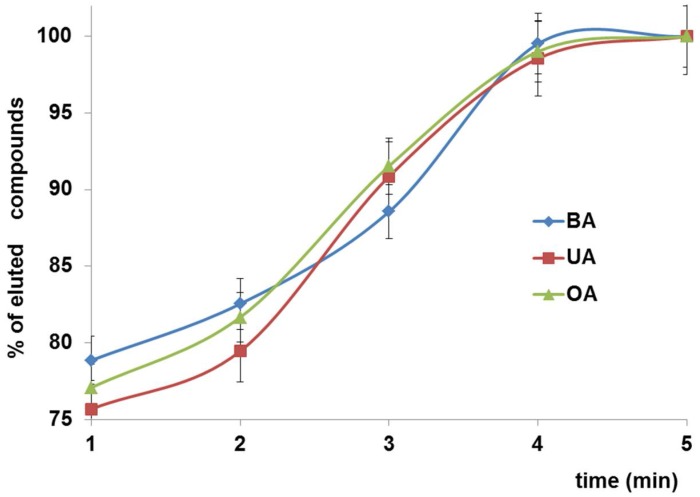
Effect of desorption time on percentage of eluted analytes.

Effect of time on desorption of analytes from sorbent is presented on Figure 8. Only slight differences of desorption kinetics between investigated compounds were noted. The amount of eluted analytes increased up to 4 min and then a plateau was observed.

### 3.3. Application of Si-PANI Sorbent for Pretreatment of Plant Material

Based on the conducted experiments, the optimal conditions for d-SPE of triterpenic acids were as follows: Si-PANI (+) impregnated with water, 3 min of extraction time, 2 mL of methanol water mixture (1:1, *v*/*v*) as washing solution, 4 min of desorption time and methanolic solution of ammonia as elution solvent. To verify the utility of the procedure, methanolic extracts from *Viscum album* L. and *Ocimum basilicum* L. were purified with the use of the above conditions. Moreover, MSPD was also conducted to assess the application potential of Si-PANI for isolation of triterpenic acids directly from raw plant material. The conditions for MSPD were established based on optimized d-SPE procedure and were as follows: Si-PANI (+) impregnated with water, 3 min of grinding time, 2 mL of methanol water mixture (1:1, *v*/*v*) as washing solution, and methanolic solution of ammonia as elution solvent.

The amount of triterpenic acids isolated using both techniques was determined by HPLC method. The validation parameters are summarized in Table 1 and the results of quantification are presented in Table 2.

**Figure 9:**
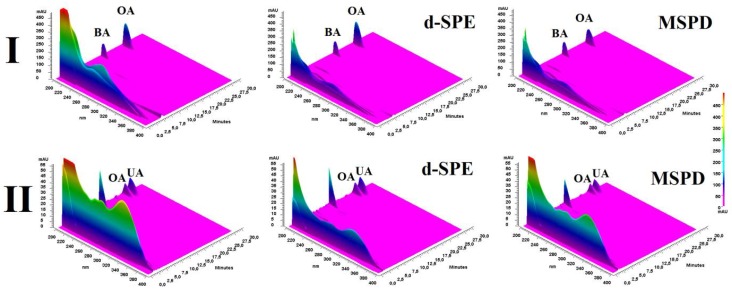
3D HPLC chromatograms of investigated plant extracts obtained with the use of various extraction methods; I-*Viscum album* L. and II-*Ocimum basilicum* L.

As can be seen, the differences between a determined amount of investigated compounds in raw and d-SPE purified extracts were relatively low (5.1 and 5.9% for BA and OA, respectively in *Viscum album* and 7.5 and 9.2% for OA and UA, respectively in *Ocimum basilicum*). Moreover, the significant reduction of the accompanying matrix comparing to raw extracts was observed in three-dimensional (3D) HPLC chromatograms (Figure 9) and this proved the utility of the proposed method for sample pre-treatment of plant extracts.

Pre clean-up of the sample is an especially essential step before chromatographic analysis. It allows us to extend the column longevity because it prevents the clogging of inter-grain spaces and pores that may led to the reduction of an active surface of stationary phase and decreasing of chromatographic system efficacy (lower theoretical plate number, resolution and peak symmetry). It also prevents the excessive increase of pressure in the chromatographic system.

The reduction of the matrix was also observed for MSPD; however, the differences of quantified analytes in raw and MSPD extract were significant (in the range of 36–42%); thus, despite the simplicity, and relatively low time-consumption, this technique may be considered only for preliminary screening studies.

In our previous study [18], two commercially available sorbents were applied for SPE of triterpenic acids; however, they were less favorable due to weak sorption (octadecyl silica) or difficult elution (aminopropyl silica). The cost of Si-PANI is slightly higher than bare silica; however, it is lower than its other modifications and Si-PANI may be an alternative for these types of sorbents.

## 4. Conclusions

In the present study, Si-PANI sorbent obtained by in situ polymerization of aniline directly on silica gel particles (Si-PANI) was applied for dispersive solid phase extraction (d-SPE) and matrix solid–phase extraction (MSPD) of plant samples before HPLC analysis of triterpenic acids. The conditions of procedures were optimized experimentally. The sorbent was protonated (+) and deprotonated to change the ionization of surface and thus, to obtain the different sorption mechanism. Both on Si-PANI (+) and Si-PANI, analytes were strongly retained; however different impregnation solutions were required (water and methanol, respectively). Due to easier elution of investigated compounds, protonated sorbent was favorable. The other established optimal conditions were as follows: 3 min of extraction time, 2 mL of methanol water mixture (1:1, *v*/*v*) as washing solution, 4 min of desorption time and methanolic solution of ammonia as elution solvent. The optimized procedure was successfully used for pretreatment of *Viscum album* L. and *Ocimum basilicum* L samples using MSPD and d-SPE. The significant reduction of the matrix was observed for both techniques; however, after MSPD, the amount of determined analytes were lower than after d-SPE and this technique may be considered only for preliminary screening studies.

## Figures and Tables

**Table 1:** Validation parameters for determination of triterpenic acids (*n* = 5).

Parameters	Oleanolic Acid	Ursolic Acid	Betulinic Acid
Concentration range	0.05–1.00 mg/mL	0.005–1.00 mg/mL	0.002–0.10 mg/mL
Correlation coefficient (r)	0.9994	0.9998	0.9999
Linear regression equation	*y* = 80,370,931*x* − 55,822	*y* = 132,468,713*x* − 13,814	*y* =101,240,680*x* − 28,874
RSD values of peak area	0.64–1.32%	0.83–1.22%	0.41–0.78%
LOD (µg/mL)	0.13	0.14	0.12
LOQ (µg/mL)	0.43	0.46	0.40

**Table 2:** The content of investigated analytes obtained with the use of various extraction methods (mg analytes/g of dried material ± SD).

Compound	*Viscum Album* L.			*Ocimum Basilicum* L.		
	Without Purification	d-SPE	MSPD	Without Purification	d-SPE	MSPD
**BA**	0.82 ± 0.10	0.78 ± 0.04	0.57 ± 0.04	-	-	-
**OA**	6.95 ± 0.41	6.55 ± 0.30	4.81 ± 0.21	0.69 ± 0.09	0.64 ± 0.07	0.45 ± 0.03
**UA**	-	-	-	1.14 ± 0.11	1.04 ± 0.10	0.77 ± 0.06
